# *Notes from the Field*: Initial Public Health Response to a Measles Outbreak in a Close-Knit West Texas Community — January−February 2025

**DOI:** 10.15585/mmwr.mm7523a2

**Published:** 2026-06-18

**Authors:** Carolyn A. Crisp, Scott Milton, Chelsea S. Lutz, Kelly Northcott, Kevin McClaran, Cynthia Hernandez, Sadia Almas, Zach Holbrooks, Katherine Wells, Elizabeth Sajewski, Texas 2025 Measles Outbreak Response Team, CDC Epi-Aid Measles Response Team, Atisha Morrison, Casey Schroeder, Maria Nolen, Jennifer Gonzales, Diana Martinez, Saroj Rai, Varun Shetty, Jennifer A. Shuford, Texas 2025 Measles Outbreak Response Team, Sandi Arnold, Brooklyn Baker, Samantha Curtis, Thi Dang, Jessica Del Toro, Eric Garza, Paola Gonzalez-Colon, Rebecca Gorman, Jenna Harlan, Elise Huebner, Anita Irving, Lydia Johnson, Michael Jost, Katie Katsounas, Bonny Mayes, Kinjal Mehta, Erica Mendoza, Karen Nunez, Bonnie Oh, Briana O’Sullivan-Kovacs, Elena Penedo, Annette Rodriguez, Daniella Rodriguez, Gretchen Rodriguez, Astrid Romero, Olivia Smith, Yan Sun, Jeff Swanson, Whitney Tillman, Shivangi Vayla, Stephen White, Cynthia Williams, Sarah Work, CDC Epi-Aid Measles Response Team, Nakia Clemmons, Thomas Filardo, Duane Hammond, Adria Mathis, Dylan Neu, Belinda Ostrowsky, Kelley Raines, Courtney Rogers, David Sugerman, Axel A. Vazquez Deida, Erika Wallender, Dennis Wang, Jonathan Yoder

**Affiliations:** ^1^Division of State and Local Readiness, Office of Readiness and Response, CDC; ^2^Texas Department of State Health Services; ^3^Epidemic Intelligence Service, CDC; ^4^Division of Viral Diseases, National Center for Immunization and Respiratory Diseases, CDC; ^5^South Plains Public Health District, Seminole, Texas; ^6^Lubbock Public Health, Lubbock, Texas; ^7^Division of Foodborne, Waterborne, and Environmental Diseases, National Center for Emerging and Zoonotic Infectious Diseases, CDC.; Texas Department of State Health Services; Texas Department of State Health Services; Texas Department of State Health Services; Texas Department of State Health Services; Texas Department of State Health Services; Texas Department of State Health Services; Texas Department of State Health Services; Texas Department of State Health Services; Texas Department of State Health Services; Texas Department of State Health Services; Texas Department of State Health Services; Texas Department of State Health Services; Texas Department of State Health Services; Texas Department of State Health Services; Texas Department of State Health Services; Texas Department of State Health Services; Texas Department of State Health Services; Texas Department of State Health Services; Texas Department of State Health Services; Texas Department of State Health Services; Texas Department of State Health Services; Texas Department of State Health Services; Texas Department of State Health Services; Texas Department of State Health Services; Texas Department of State Health Services; Texas Department of State Health Services; Texas Department of State Health Services; Texas Department of State Health Services; Texas Department of State Health Services; Texas Department of State Health Services; Texas Department of State Health Services; Texas Department of State Health Services; Texas Department of State Health Services.; CDC; CDC; CDC; CDC; CDC; CDC; CDC; CDC; CDC; CDC; CDC; CDC; CDC.

**Summary**

**What is already known about this topic?**

Measles is a highly contagious respiratory virus that can cause serious illness. A measles outbreak occurred in Texas during January–August 2025.

**What is added by this report?**

During January 29–February 28, 2025, Texas reported 207 confirmed measles cases, primarily among members of a close-knit west Texas community. Most cases occurred among unvaccinated persons or those with unknown vaccination status. Measles, mumps, and rubella (MMR) vaccine and measles testing clinics were offered; however, community members were hesitant to interact with public health and health care systems, and MMR vaccine acceptance was low. Educational materials on measles and measles prevention were developed and distributed.

**What are the implications for public health practice?**

In challenging community contexts, public health messaging intended to limit viral transmission and severe health outcomes could supplement standard control measures, including advising persons with suspected measles to avoid contact with other persons to prevent transmission and to seek medical care promptly.

## Introduction

On January 29, 2025, the South Plains Public Health District (SPPHD) alerted the Texas Department of State Health Services (DSHS) Public Health Region 1 of an unvaccinated school-aged child with measles in Gaines County, a rural county in west Texas bordering New Mexico. Investigations during January 29–February 28, 2025, identified 207 confirmed cases^†^ (144 laboratory confirmed and 63 epidemiologically linked), predominately in a multilingual, close-knit community in Gaines County and eight nearby counties. This report describes barriers to implementing public health interventions during the initial phase of the outbreak. This activity was reviewed by CDC, deemed not research, and conducted consistent with applicable federal law and CDC policy.^§^

## Investigation and Outcomes

### Characteristics of Persons with Measles

Among 207 measles cases in Texas residents reported during January 29–February 28, approximately two thirds (143; 69%) were reported in Gaines County; the remaining 64 (31%) occurred in eight nearby counties (Table). The median age of persons with measles was 7 years (range = 0 days–57 years), and 115 (56%) cases occurred in females. Among 22 females of childbearing age (15–44 years) with measles, two (10%) were pregnant. Among reported cases during this time, 38 (18%) patients were hospitalized (*1*). On February 26, 2025, an unvaccinated school-aged child with measles died. Overall, 348 clinical specimens were collected for confirmatory testing and viral genotype analysis; among 106 (30%) that were successfully genotyped, all were genotype D8 (*2*).

**TABLE:** Number and percentage of persons with measles, by demographic characteristics, hospitalization status, and vaccination status — west Texas measles outbreak, January 29−February 28, 2025

Characteristic	No. (%)
Total	207 (100)
**County of residence**	
Gaines	143 (69)
Terry	35 (17)
Yoakum	9 (4)
Dawson	9 (4)
Dallam, Martin, Ector, Lubbock, and Lynn*	11 (5)
**Age group, yrs**	
0–4	68 (33)
5–17	105 (51)
≥18	33 (16)
Unknown	1 (<1)
**Sex**	
Female	115 (56)
Pregnant, aged 15–44 yrs (n = 22)	2^†^ (9)
Male	92 (44)
**Hospitalization status**	
Hospitalized	38 (18)
Not hospitalized	169 (82)
**No. of MMR vaccine doses received**	
None or unknown^§^	201 (97)
1^¶^	2 (1)
≥2^¶^	4 (2)

**Abbreviation**: MMR = measles, mumps, and rubella.

* Because each of these counties reported fewer than five cases, they were combined to avoid potential identification of persons.

^†^ Gestational ages were 29 weeks and 35 weeks.

^§^ Disaggregating unvaccinated persons from those with unknown vaccination status was not possible because the Texas Immunization Registry requires explicit consent by law (i.e., is an opt-in registry) to enroll. Unknown vaccination status includes persons who reported having received MMR vaccine but whose vaccination status could not be verified and those who could not recall if they had received MMR vaccine. Unvaccinated status includes persons with no documented doses of MMR vaccine >14 days before symptom onset.

^¶^ Review of the available information indicated that these persons received ≥1 age-appropriate dose of MMR vaccine >14 days before symptom onset or best available proxy date (if the date of rash was not available, one of the following dates was used, in this order: symptom onset date, specimen collection date, hospital admission date, or date reported to the region). Measles Outbreak – August 12, 2025 | Texas DSHS

### Vaccination Status of Persons with Measles and Local Vaccination Coverage

Among 207 persons with confirmed measles, 201 (97%) had no documentation of receipt of measles, mumps, and rubella (MMR) vaccine or their vaccination status was unknown; six (3%) had received ≥1 vaccine dose. In the outbreak area, county MMR vaccination coverage among kindergarteners during the 2024–25 school year ranged from 77.3% to 94.6%, compared with 93.2% in Texas overall. To prevent in-school transmission (and be consistent with Texas law), public health professionals recommended that school administrators ask students not to return to school for 21 days after a measles exposure if they did not have documented evidence of immunity (receipt of ≥2 valid MMR vaccine doses, documented prior infection, or positive antimeasles immunoglobulin G titers). Children attending unaccredited private schools or those who were homeschooled would not be included in this policy, potentially leading to ongoing school-based transmission.

### Response to Distribution of Measles Information and Prevention Materials

SPPHD and DSHS developed and distributed culturally appropriate and community-informed resources describing measles disease and prevention strategies, including school guidance, information for school nurses and parents, and vaccination and testing clinic locations. CDC helped translate materials into relevant languages. During January 29–February 28, public health education focused on the importance of vaccination and symptom recognition. In counties with evidence of ongoing measles transmission, an early dose of MMR vaccine for infants aged 6–11 months and a second MMR vaccine dose for adults who had received only 1 dose were recommended. Thirty-three MMR vaccination clinics for persons aged ≥6 months and 16 measles testing clinics were held in DSHS Public Health Region 1. Despite these measures, vaccine acceptance was low; approximately 275 MMR vaccine doses were administered. Understanding transmission dynamics in the affected community was difficult because many persons interviewed during case investigations declined to provide enough information to enable follow-up and an exposure assessment (e.g., names of household members [often in large household units], contacts, or exposures).

## Preliminary Conclusions and Actions

Low rates of MMR vaccination and measles testing and reluctance among community members while being interviewed during case investigations presented substantial challenges during the initial weeks of this measles outbreak. SPPHD and DSHS contacted trusted community members to better understand perspectives of the community. Many community members described their lack of trust in outside institutions and their reluctance to engage with public health and health care systems overall, based on an ethos within the community that prioritized maintaining independence from outside institutions and seeking solutions from within the community. This perspective complicated implementation of standard measles control measures and hampered epidemiologic investigations. Therefore, many measles cases likely remained unreported.

Decreases in measles vaccination coverage worldwide have increased the risk for larger measles outbreaks, especially in undervaccinated communities (About Measles | CDC). On August 18, 2025, the Texas measles outbreak was declared over, having comprised 762 confirmed cases, 99 hospitalizations, and a second measles-associated death. Although the source of this outbreak remains unknown, internationally imported cases have been associated with outbreaks among other close-knit U.S. communities with low vaccination coverage (*3*,*4*). High population coverage with 2 MMR vaccine doses is the most effective public health intervention for preventing measles (Measles Vaccination | CDC).

This outbreak highlights several challenges associated with encouraging vaccination, testing, education, and other interventions to limit disease severity and spread in certain communities. Early in a measles outbreak response, public health partnerships with trusted community members might help guide the development of culturally appropriate educational materials to support public health interventions. In challenging community contexts, public health messaging intended to limit viral transmission and severe health outcomes could supplement standard control measures, including advising persons with suspected measles to avoid contact with others to prevent transmission and to seek medical care promptly.

## Footnotes

† A confirmed measles case was defined as an acute, febrile rash illness (temperature can be <101°F [38.3 °C] or subjective and rash of <3 days’ duration) that is laboratory confirmed or epidemiologically linked to a laboratory-confirmed case, or a febrile rash illness in a person living in or visiting (within the past 21 days) any of the following outbreak counties as of February 28, 2025: Dawson, Gaines, Lynn, Martin, Terry, and Yoakum (Measles Outbreak Case Definition | Texas DSHS). Laboratory-confirmed cases were those among persons who received a positive wild-type measles test result using one of the methods described in the 2025 Texas DSHS Epi Case Criteria Guide.

§ 45 C.F.R. part 46, 21 C.F.R. part 56; 42 U.S.C. Sect. 552a; 44 U.S.C. Sect. 3501 et seq.
